# Gap junction mediated miRNA intercellular transfer and gene regulation: A novel mechanism for intercellular genetic communication

**DOI:** 10.1038/srep19884

**Published:** 2016-01-27

**Authors:** Liang Zong, Yan Zhu, Ruqiang Liang, Hong-Bo Zhao

**Affiliations:** 1 Department of Otolaryngology, University of Kentucky Medical Center, 800 Rose Street, Lexington, KY 40536 U.S.A.

## Abstract

Intercellular genetic communication is an essential requirement for coordination of cell proliferation and differentiation and has an important role in many cellular processes. Gap junction channels possess large pore allowing passage of ions and small molecules between cells. MicroRNAs (miRNAs) are small regulatory RNAs that can regulate gene expression broadly. Here, we report that miRNAs can pass through gap junction channels in a connexin-dependent manner. Connexin43 (Cx43) had higher permeability, whereas Cx30 showed little permeability to miRNAs. In the tested connexin cell lines, the permeability to miRNAs demonstrated: Cx43 > Cx26/30 > Cx26 > Cx31 > Cx30 = Cx-null. However, consistent with a uniform structure of miRNAs, there was no significant difference in permeability to different miRNAs. The passage is efficient; the miRNA level in the recipient cells could be up to 30% of the donor level. Moreover, the transferred miRNA is functional and could regulate gene expression in neighboring cells. Connexin mutation and gap junctional blockers could eliminate this miRNA intercellular transfer and gene regulation. These data reveal a novel mechanism for intercellular genetic communication. Given that connexin expression is cell-specific, this connexin-dependent, miRNA intercellular genetic communication may play an important role in synchronizing and coordinating proliferation and differentiation of specific cell types during multicellular organ development.

Genetic communication between cells is required for many physiological and pathological cellular processes, such as synchronization and coordination of cell proliferation and differentiation in tissue homeostasis and during organ development^1^. However, the underlying mechanisms are poorly understood. Intercellular transfer of RNAs and nucleotides was proposed early in 1970s^2^. It has been reported that RNAs can be transported among cells by microvesicles through exocytosis and endocytosis via extracellular space^1^,^3^. However, this microvesicle-based RNA intercellular transport is inefficient due to unavoidable dilution in the extracellular space. It is estimated that only very small fraction (~ 0.7%) of the released RNAs can be absorbed to re-enter into cells^3^. Moreover, this type of intercellular transport is less selectable to achieve cell-specific delivery, which is extremely important for controlling and coordinating the proliferation and differentiation of specific cell types in multicellular organ development.

Gap junctions are intercellular channels and represent the only intercellular conduit that possesses large pore size (1.0–1.5 nm) and allow passage of ions and small molecules from one cell interior to another directly^4^. MicroRNAs (miRNAs) are small non-coding RNAs, which can modulate gene expression widely by affecting the translation of mRNAs to proteins and inducing mRNA target decay^5^,^6^,^7^,^8^. A miRNA is single-stranded and ~21 nucleotides long^5^,^6^, forming a linear molecule with a diameter of ~1.0 nm^3^,^9^, which is in the same order of the gap junction channel pore size. Recently, it has been reported that miRNAs can be exchanged between tumor cells in a gap junction-dependent manner^10^,^11^,^12^. However, it is unclear whether this is a general phenomenon and whether the exchanged miRNAs are functional. Detailed information also remains unclear, since gap junctional coupling and connexin expression in these tumor-cells have not been well characterized. In this study, we used connexin-defined cell lines and found that miRNAs can pass through gap junctions to regulate gene expression in neighboring cells. This gap junction-mediated miRNA intercellular transfer and gene regulation provides a novel mechanism for intercellular genetic communication.

Preliminary reports of this work have been presented in abstract forms^13^,^14^.

## Results

### Transfer of miRNAs between cells via gap junctions

**Figure 1:**
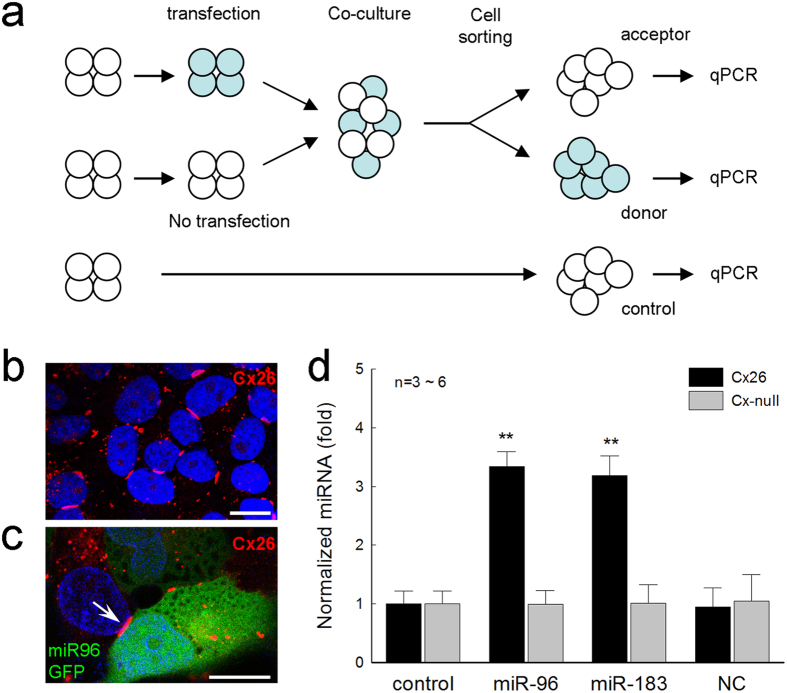
Intercellular transfer of miRNAs via gap junctional coupling. (**a**) Schematic drawing of experimental procedure of miRNA intercellular transfer assessed by quantitative RT-PCR (qPCR). Non-transfected cells and miRNA GFP transfected cells are co-cultured for 36–48 hr and sorted by fluorescence-activated cell sorting (FACS). No transfection, no co-culture cells served as a control group. (**b**) Immunofluorescent staining of Cx26 cell line for Cx26. Cell nuclei are visualizes by DAPI staining (blue). Gap junctional plaques (red) between cells are visible. (**c**) Formation of gap junctions between miR-96 GFP transfected (GFP+) cells and non-transfected (GFP−) cells in Cx26 cell line after co-culture for 36 hr. An arrow indicates a gap junctional plaque formed between GFP + and GFP− cells. (**d**) Expression of mouse miRNAs in the non-transfected cells after co-culture with cells transfected with miR-96, miR-183, and non-miRNA construct (NC) GFP vectors in Cx26 and Cx-null cell lines. The expression levels were normalized to the control group (no co-culture cells) in each cell line. **P < 0.001, one-way ANOVA with a Bonferroni correction. Scale bars: 10 μm.

MicroRNAs have a uniform structure and similar size. Since miR-96 and miR-183 are predominant miRNAs in the inner ear and play an important role in the inner ear development and hearing^15^, we selected miR-96 and miR-183 to test in this study. In order to test whether miRNAs can pass through gap junctions, we used connexin expression defined human HeLa cell lines. In each cell line, two groups of cells were transfected with mouse miRNA with GFP and empty non-miRNA construct vector with GFP (NC-GFP), respectively (Fig. 1a and Supplementary Fig. S1). Then, transfected (GFP+) cells were mixed with non-transfected (GFP−) cells and co-cultured allowing forming gap junctions between them. After co-culture for 36–48 hr, gap junctions between them are visible (Fig. 1c) and the co-cultured transfected (GFP+) cells and non-transfected (GFP−) cells were separated by fluorescence-activated cell sorting (FACS). In each cell line, including Cx-null cell line, the non-transfected cells without co-culture served as a control group (Supplementary Fig. S1). Figure 1d shows that the levels of miRNA expression in the non-transfected cells were significantly increased after co-cultured with miRNA-transfected cells in the Cx26 cell line. The expression levels of mouse miR-96 and miR-183 in the non-transfected cells in the Cx26 cell line were increased by more than 3-fold in comparison with those in the control no co-culture group (P < 0.001, one-way ANOVA with a Bonferroni correction). However, the expression levels of miR-96 or miR-183 in the non-transfected (GFP−) cells after co-culture and cell sorting in the Cx-null cell line were 0.99 ± 0.23 and 1.01 ± 0.32 (fold), respectively and remained at the background level (Fig. 1d). In comparison with that at the control group, they were not significantly increased (P = 0.91, one-way ANOVA), indicating that there was also no apparent contamination during cell sorting.

Moreover, after co-culture with NC-GFP transfected cells, the expression of miR-96 in the non-transfected cells was also not increased (Fig. 1d). The expression levels of miR-96 in the non-transfected cells after co-cultured with NC-GFP transfected cells in the Cx26 cell line and Cx-null cell line were 0.94 ± 0.32 and 1.04 ± 0.45 (fold), respectively (Fig. 1d). In comparison with control group, they were not significantly increased (P = 0.34, one-way ANOVA). This further indicates that there was no significant interfering of endogenous miRNAs in measurement.

Fig.1d also shows that the expression levels of miR-96 and miR-183 in the non-transfected cells after co-culture with each miRNA transfected cells in the Cx26 cell line were similar, and were increased by 3.34 ± 0.25 and 3.18 ± 0.34 (fold), respectively. There was no significant difference between increments in miR-96 and miR-183 expressions (P = 0.89, one-way ANOVA).

**Figure 2:**
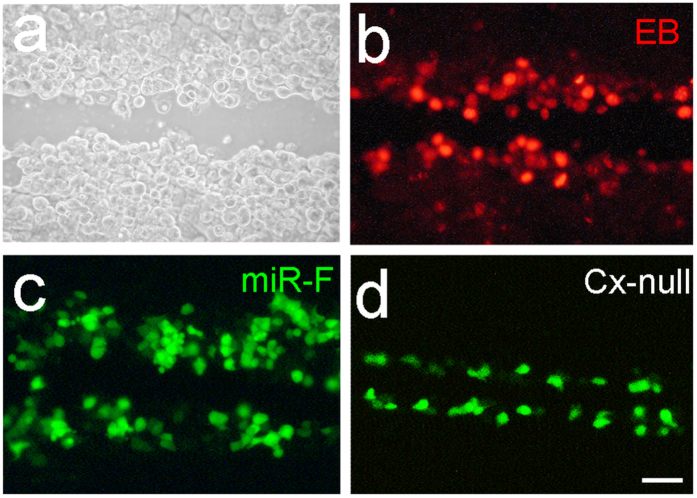
Gap junction mediated intercellular transfer of miRNAs assessed by scrape-loading assay. (**a–c**) Intercellular diffusion of fluorescence-tagged miRNA (miR-F) and dye ethidium bromide (EB) in the Cx43 cell line. The images were captured after 30 min for scrape-loading. (**d**) No diffusion of the miR-F is visible in the Cx-null cell line. Scale bar: 25 μm.

We also used a fluorescence-tagged miRNA (miR-F) to assess miRNA intercellular transfer. Figure 2 shows that miR-F could pass through gap junctions to neighboring cells in connexin cell lines in scrape-loading. However, there was no diffusion in the Cx-null cell line, in which the miR-F was limited to the scraped cells at the scrape-edge (Fig. 2d).

### Intercellular transfer of miRNAs is connexin-dependent

**Figure 3:**
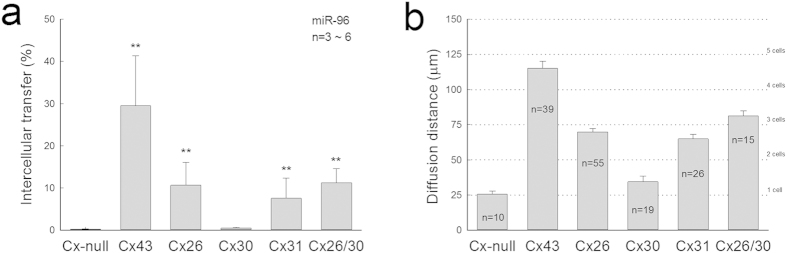
Connexin-dependence of miRNA intercellular transfer. (**a**) The percentage of miR-96 transfer calculated from the expression levels of non-transfected cells vs miRNA-transfected cells in various connexin cell lines after co-culture. **P < 0.001, one-way ANOVA with a Bonferroni correction. (**b**) Diffusion of miR-F in various connexin cell lines in scrape-loading assay. Data are represented as mean ± SD.

We further quantitatively analyzed and compared intercellular transfer of miRNAs in different connexin cell lines. Figure 3a shows the percentage of intercellular transport, which was calculated by the level of miRNAs in the non-transfected (recipient) cells *vs* the level of miRNAs in the transfected (donor) cells, measured from different connexin cell lines after co-culture. The percentage of miR-96 in the non-transfected cells was 29.9 ± 11.9, 10.6 ± 5.47, 7.60 ± 4.80, 11.3 ± 3.33, 0.50 ± 0.16, and 0.24 ± 0.10% in Cx43, Cx26, Cx31, Cx26/30, Cx30, and Cx-null cell lines, respectively. Cx43 channels demonstrated higher permeability to miRNAs, whereas Cx30 channels were little permeable to miRNAs. Consistent with qPCR measurement, the scrape-loading assay also shows that the diffusion of miR-F in the Cx43 cell line was broader and reached the 4^th^–5^th^ cell order from the edge of the scrape (Fig. 3b), whereas the diffusion of miR-F in the Cx30 cell line was minimal and limited to the 1^st^ cell order at the scraped edge. The diffusion of miR-F demonstrated the same order as measured by qPCR: Cx43 > Cx26/30 > Cx26 > Cx31 > Cx30 = Cx-null (Fig. 3b). However, there was no significant difference in the diffusion of dye ethidium bromide (EB) among these connexin cell lines (Supplementary Fig. S2), indicating that functional expression of connexins in these connexin over-expressed cell lines was similar.

### Blockage of miRNA intercellular transfer by gap junctional blockers

**Figure 4:**
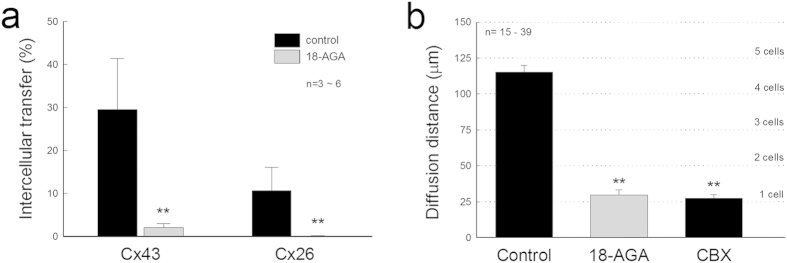
Blockage of miRNA intercellular transfer by gap junction channel blockers. (**a**) The intercellular transfer of miR-96 in Cx43 and Cx26 cell lines is significantly reduced by application of 50 μM 18-AGA during the co-culture. (**b**) Gap junction channel blockers block the intercellular diffusion of miR-F in the Cx43 cell line. The loaded miR-F is limited to the scraped cells in the presence of 18-AGA (50 μM) or carbenoxolone (CBX, 0.1 mM). Data are represented as mean ± SD. **P < 0.001, one-way ANOVA with a Bonferroni correction.

Gap junctional blockers could block miRNA intercellular transfer. Figure 4 shows that application of 50 μM 18α-glycyrrhetinic acid (18-AGA) significantly reduced miRNA intercellular transfer. The miR-96 levels in the non-transfected cells in the Cx43 and Cx26 cell lines were significantly reduced from 29.9 ± 11.9 and 10.6 ± 5.47% to 2.12 ± 0.87 and 0.12 ± 0.11% (P < 0.001, one-way ANOVA with a Bonferroni correction), respectively, at the background level (Fig. 4a). Application of 50 μM 18-AGA or 0.1mM carbenoxolone (CBX) also blocked miR-F diffusion in the Cx43 cell line in the scrape-loading assay; there was no apparent intercellular diffusion (Fig. 4b).

### Disruption of miRNA intercellular transfer by connexin mutation

**Figure 5:**
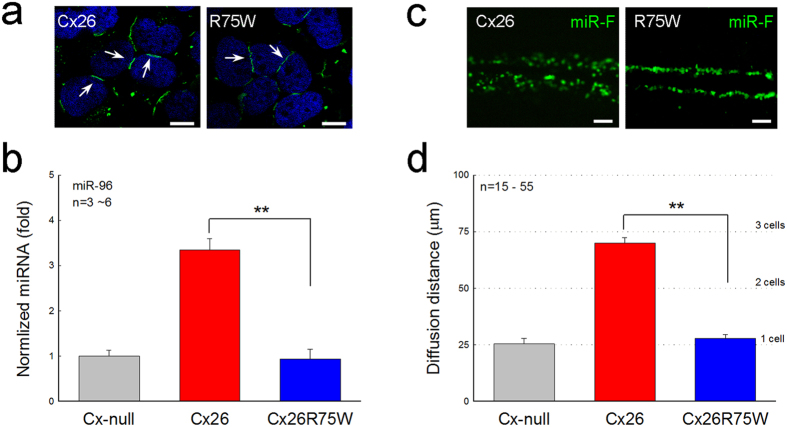
No miRNA intercellular transport in the Cx26 R75W mutant cell line. (**a**) Immunofluorescent staining for Cx26 in Cx26 and Cx26 R75W cell lines. White arrows indicate gap junctional plaques between cells in both the Cx26 cell line and the Cx26 R75W cell line. (**b**) The expression of miR-96 in non-transfected cells is increased after co-culture with miR-96 transfected cells in the Cx26 cell line but not in the Cx26 R75W cell line. (**c,d**) There is no intercellular diffusion of miR-F in the Cx26 R75W cell line in the scrape-loading assay. Data are represented as mean ± SD. **P < 0.001, t-test. Scale bars: 10 μm in (**a**), 25 μm in (**c**).

The permeability to miRNAs could also be disrupted by connexin mutation. Deafness -associated Cx26 p.R75W mutant can express at the plasma membrane forming gap junctional plaques between cells (Fig. 5a) but has no permeability and transjunctional conductance^16^,^17^. In the Cx26 R75W cell line, the expression of miR-96 in the non-transfected cells after co-culture was not increased and remained at the background level (Fig. 5b). The scrape-loading assay also showed that there was no intercellular diffusion of miR-F in the Cx26 R75W cell line (Fig. 5c,d); the loaded miR-F was restricted at the scrape edge (Fig. 5c) as shown in the Cx-null cell line in Fig. 2d.

### Gene regulation in neighboring cells

**Figure 6:**
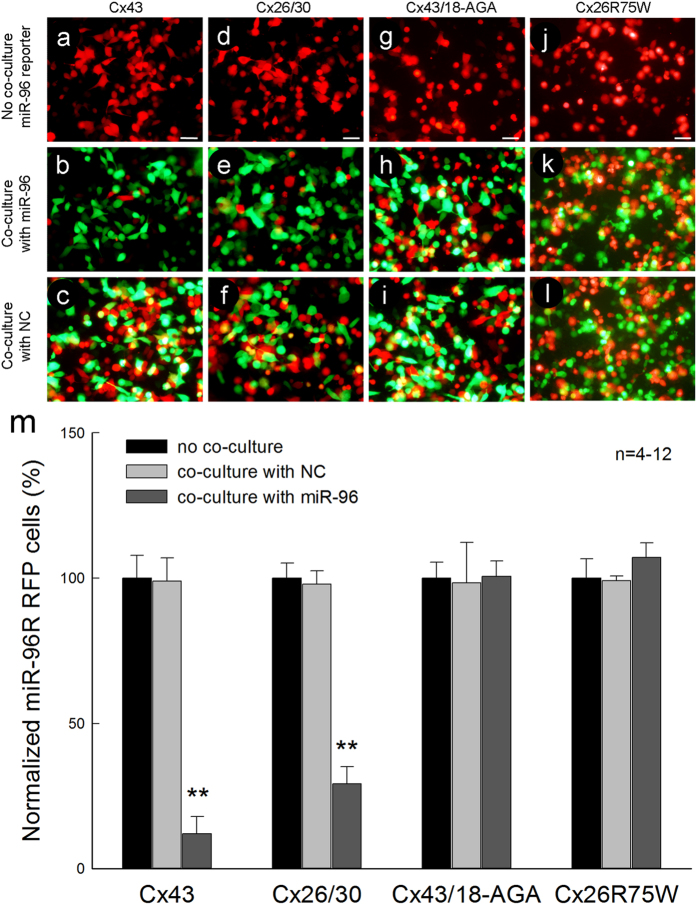
Silencing of gene in neighboring cells by transferred miRNAs. (**a–f**) Expression of RFP miR-96 reporter (RFP-miR-96R) in Cx43 and Cx26/30 cell lines is inhibited after co-culture with miR-96 GFP transfected cells but not co-culture with NC GFP transfected cells. (**g–i**) Application of 18-AGA (50 μM) restored RFP-miR-96R expression in the Cx43 cell line in co-culture with miR-96 GFP transfected cells. (**j–l**) There is no inhibition in RFP-miR-96R expression in the Cx26 R75W cell line when co-cultured with miR-96 GFP transfected cells. Scale bars: 50 μm. (**m**) Quantitative analyses of silencing of RFP-miR-96R reporter expression in different connexin cell lines after co-culture with miR-96 and NC transfected cells. In each cell line, the numbers of RFP-positive cells in co-culture with miR-96 GFP and NC GFP transfected cells were normalized to the number of RFP-positive cells in the no co-culture control group. **P < 0.01, one-way ANOVA with a Bonferroni correction.

We further tested whether the transported miRNAs are functional and can regulate gene expression in neighboring cells. We used mouse miR-96 reporter fused red fluorescence protein (RFP) (RFP-miR-96R). So, RFP expression can be specifically silenced by mouse miR-96. Mouse miR-96 (miR-96-GFP) and reporter (RFP-miR-96R) were separately transfected into cells and co-cultured. Figure 6a–f shows that after co-culture with miR-96-GFP transfected cells, RFP expression in RFP-miR-96R transfected cells was significantly reduced. In comparison with RFP-positive cells in the no co-culture control group (Fig. 6a,d), the RFP-positive cells in co-culture groups in Cx43 and Cx26/30 cell lines were significantly reduced to 12.0 ± 6.0% and 29.1 ± 5.97% (P < 0.001, t-test), respectively (Fig. 6b,e,m). However, RFP-miR-96R expressions in co-culture with NC-GFP empty vector transfected cells in the same Cx43 and Cx26/30 cell lines were similar to those in no co-culture control groups (Fig. 6c,f,m). The expression of RFP-miR-96R in the Cx26 R75W cell line was also not reduced and not changed when co-cultured with miR-96-GFP transfected cells (Fig. 6j–m). Moreover, silencing of RFP-miR-96R expression in co-culture with miR-96 transfected cells in Cx cell line could be blocked by gap junctional blockers. Fig. 6g–i show that application of 50 μM 18-AGA restored the expression of RFP-miR-96R in co-culture with miR-96 transfected cells in Cx43 cell line as the same as that in the control group (Fig. 6m).

## Discussion

**Figure 7:**
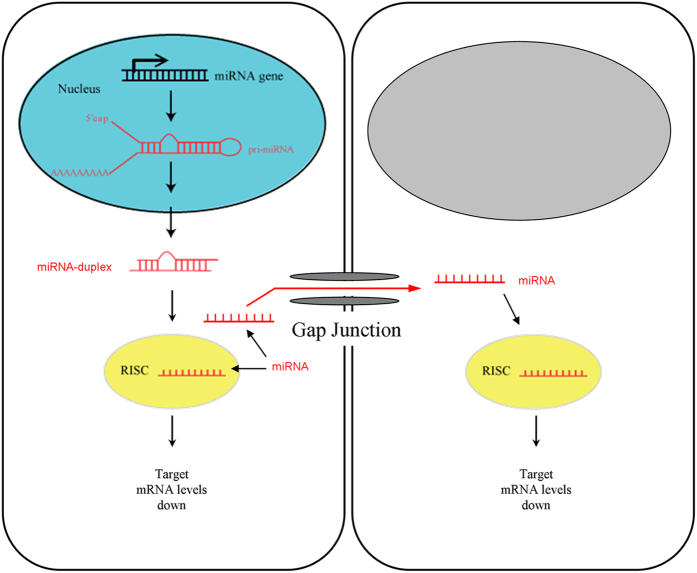
Schematic drawing of gap junction mediated miRNA intercellular communication and gene regulation.

Gap junctions provide a direct intracellular conduit between cells, allowing exchanging ions and small substances up to molecular weight of 1.5 kDa^4^. Gap junction channels are aqueous channels that can enable intercellular electrical communication. Gap junction channels can also exchange metabolic signaling molecules, such as calcium, cAMP/cGMP, and IP_3_, among cells to coordinate cell metabolic processes for intercellular metabolic communication^4^. In this study, we found that miRNAs can pass through gap junctional channels to regulate gene expression in neighboring cells (Figs 1, 2, 3 and 6), suggesting that gap junctions can also play important intercellular genetic communication to synchronize and coordinate gene expressions among cells (Fig. 7). Gap junctions exist in almost all types of cells and organs. Moreover, gap junctions in the organs usually form functional networks. Thus, this gap junction-mediated miRNA intercellular gene regulation could provide a novel mechanism for synchronization and coordination of gene expression among a broad range of cells.

This gap junction-mediated miRNA intercellular communication is also efficient. In comparison with microvesicle-based RNA intercellular transport that the percentage of intercellular transport is estimated to be only ~0.7%^3^, the percentage of intercellular transport of miRNAs by gap junctions was much higher (Fig. 3). Except Cx30, the percentage of intercellular transport of miRNAs in the tested connexin cell lines was greater than 8% (Fig. 3a). For Cx43, which is expressed robustly in many cell types^4^, the percentage of intercellular transport could be up to 30% (Fig. 3a). Furthermore, a single miRNA can reduce the stability of hundreds of unique mRNAs and can repress the production of hundreds of proteins^18^,^19^,^20^, and also there was no apparent difference in permeability to different miRNAs (Figs 1d,2). Thus, this gap junction mediated miRNA intercellular communication can provide an efficient and widespread mechanism to coordinate gene regulation and function among cells.

In the experiments, we also found that this gap junction mediated miRNA intercellular transfer is connexin-dependent (Fig. 3). In the tested connexin cell lines, the permeability to miRNAs demonstrates a following order: Cx43 > Cx26/30 > Cx26 > Cx31 > Cx30 = Cx-null; Cx43 has high permeability to miRNAs, while Cx30 is little permeable to miRNAs (Fig. 3). This is consistent with previous reports that Cx30 channels are impermeable to negatively charged molecules^21^,^22^, since all nucleotides including miRNAs are anionic at physiological pH. This difference of permeability in different connexins may also have an important implication that this connexin-dependent miRNA intercellular transfer and gene regulation can provide a cell-specific intercellular genetic communication, because connexin expression is cell-specific^4^. In particular, such cell-specific intercellular genetic communication can have an important role in synchronizing and coordinating proliferation and differentiation of specific cell types in multicellular organ development.

Indeed, gap junction mediated intercellular communication plays a critical role in the inner ear development. Cx26 and Cx30 are predominant isoforms co-expressed in the cochlea^23^,^24^. It has been found that Cx26 deficiency can induce cochlear developmental disorders^24^,^25^,^26^,^27^,^28^, whereas deletion of co-expressed Cx30 displayed normal cochlear development^24^,^28^. Recently, we further found that Cx26 deletion but not Cx30 deletion can disrupt miRNA intercellular communication in the cochlea with cochlear developmental disorders^28^. In the experiment we found that Cx26 p.R75W mutation disrupted miRNA intercellular communication (Figs 5 and 6). Cx26 mutation p.R75W can also induce cochlear developmental disorders and deafness^24^,^29^. Furthermore, we previously reported that Cx26 in the cochlea is responsible for gap junction-mediated anionic molecule permeability and metabolic communications^30^. Thus, Cx26 may also play an important role in the intercellular genetic communication in the cochlea. Cx26 deficiency impairs miRNA intercellular communication in the inner ear and inner ear development^28^, and eventually leads to congenital deafness as previously reported^23^,^25^,^26^.

MicroRNAs also have broad function and can play an important role in DNA repair, apoptosis, oxidative stress response, immune response, and organ development^7^,^31^,^32^. To date, approximately 300 conserved miRNA families and thousands of additional poorly conserved miRNAs have been identified in mammals. Approximately two thirds of all human protein-coding genes are conserved targets of miRNAs^6^,^7^. Moreover, gap junctions extensively exist in almost all cell types and organs. Also, miRNAs can survive and function for several hours and even days^6^. Thus, this miRNA-mediated genetic intercellular communication may offer a new approach to the development of miRNA-based, gap junction-mediated gene therapies, as suggested by previous studies using other small regulatory RNAs, such as siRNAs, for gene therapies^9^,^33^,^34^,^35^.

## Materials and Methods

### miRNA expression vectors

Mouse miR-96 and miR-183 GFP vectors were constructed by Lentivector-based microRNA Precursor Constructs (PMIRHxxPA-1, System Bioscience) following manufacturer’s instructions. Mouse miR-96 and miR-183 were cloned by using the following primers: miR-183-F: 3′-AAG GCA GCT GAC CCC TCT GC-5′; miR-183-R: 3′-GAA CAG GCC CTC TGG GGA AG-5′ and miR-96F: 3′-GGC CTG TTC CAG TAC CAT CT-5′; miR-96-R: 3′-GCC CAG CTC GGA TTG CCC AG-5′. Mouse (C57BL/6J) genomic DNA was used as template. PCR product was cloned into pCMV-MCS-EF1-copGFP Lentiviral plasmid (Systems Biosciences, MountainView, CA), and was verified by sequencing. The empty non-miRNA construct (NC) vector, which contains GFP, was used as an internal control. The mouse miR-96 reporter was constructed by cloning of perfectly matched reverse complimentary sequence of mouse miR-96 into ptdTomato-C1 vector (cat. #632533, Clontech) fused with red fluorescent protein (RFP) and can be specifically bound by mouse mR-96 to silence RFP expression.

### Connexin HeLa cell line culture and miRNA transfection

Cx26, Cx30, Cx26/30, Cx31, Cx43, Cx26 R75W, and connexin-null (Cx-null) human HeLa cell lines were obtained from Dr. Yum’s laboratory and Dr. Willecke’s laboratory. These connexin and Cx-null cell lines were established by transfection with defined connexin(s) and fully characterized in previous studies^36^,^37^. These connexin-defined and Cx-null HeLa cells were cultured in DMEM, which contains ~2 mM Ca^++^ and ~1 mM Mg^++^, (Gibco BRL, Life Technologies, USA) with 10% fetal bovine serum and 100U/ml penicillin at 37 °C in a 5% CO_2_ incubator. For miRNA transfection, cells were passed by trypsin-EDTA and re-seeded with a density of 100,000 cells per well in a 24-well plate and incubated overnight. The medium was then replaced with the fresh DMEM plus 10% FBS and a transfection reaction mixture, which contained OPTI-MEM medium, Lipofectamine 2000 (Invitrogen) and the miRNA plasmid, following manufacturer’s instructions. After 18–24 hours, successful transfectants were verified under the fluorescent microscope.

### Cell co-culture and fluorescence-activated cell sorting (FACS)

In each experiment, the selected connexin cell lines and the control Cx-null cell line were cultured in parallel. For each cell line, the cultured cells were divided into 4 groups: two groups were transfected with miRNA GFP vectors and empty non-miRNA construct (NC) GFP vectors, and two groups had no transfection (Supplementary Fig. S1). After 18–24 hr, the cultured cells were disassociated with trypsin, and the transfected cells were co-cultured with non-transfected cells at 1:1 ratio, allowing formation of gap junctions between them (Fig. 1c). After co-culture for 36–48 hr, the co-cultured cells were disassociated by trypsin, washed with culture medium for 2-3 times, and sorted by iCyt Synergy sorter system (a Becton-Dickinson LSRII). Transfected (GFP+) cells and non-transfected (GFP−) cells were separately collected for miRNA measurement (Fig. 1a and Supplementary Fig. S1).

### miRNA extraction and quantitative PCR measurement

As we previously reported^28^, miRNAs in sorted GFP(+) cells, GFP(−) cells, and no co-culture cells (1 × 10^6^ cells in each group) were extracted by use of mirVana miRNA Isolation Kit (AM1560, Ambion, USA) following manufacturer’s instructions. The purity and quantity of miRNA was determined by a NanoDrop ND-1000 Spectrophotometer (NanoDrop Technologies, Inc., Rockland, DE). Then, as we previously reported^28^, miRNAs were converted to cDNA using TaqMan^®^ MicroRNA Reverse Transcription Kit (#4366596, Applied Biosystems, CA, USA) with corresponding mouse-specific miRNA reverse transcription templates according to manufacturer’s instructions, and measured by use of MyiQ real-time PCR detection system (Bio-Rad Laboratories) with TaqMan^®^ MicroRNA Assay (Applied Biosystems, CA, USA). An internal standard U6 snRNA (#001973, Applied Biosystems, CA) was used as an internal control. The relative quantity of miRNA expression was calculated from the standard curve and normalized to the amount of the internal standard U6 snRNA^28^. The miRNA levels in both transfected (GFP+) group and non-transfected (GFP−) group were calculated. The percentage of miRNA intercellular transport was further calculated by the miRNA level in the non-transfected (GFP−) cells *vs* the miRNA level in the transfected (GFP+) cells sorted from the same co-culture group.

### Scrape-loading for assessing the gap junctional permeability to miRNAs

For scrape-loading to assess the permeability of gap junctional channels to miRNA, a fluorescence-tagged miRNA (miR-F), which is constructed by a 25 nt miRNA (5′-CCT CTT ACC TCA GTT ACA ATT TATA-3′) labeled with carboxyfluorescein on its 3′ end (Gene Tools, Inc. OR), was used as we previously reported^28^. This miR-F was proven to be not hybridized or degraded and also had no fluorescent tag removal in the cytoplasm^38^,^39^. For performance of scrape-loading, cells were grown to confluence and incubated in 100 μM miR-F. Parallel lines were cut by a razor blade. After 30 min, cells were washed with HBSS and the diffusion of miR-F was imaged. The distance from the scrape edge to the point where the average fluorescence intensity dropped to 1.5X the background intensity were measured by NIH imageJ software (NIH, Bethesda, MD, USA).

### Immunofluorescent staining

The immunofluorescent staining was performed as previously reported^23^. The cultured cells were fixed with 4% paraformaldehyde for 30 min and washed with PBS. After 30 min of incubation in a blocking solution (10% goat serum and 1% BSA in PBS) with 0.1% Triton X-100, the cultured cells were incubated with monoclonal mouse anti-Cx26 (1: 400, Cat#33-5800, Invitrogen) in the blocking solution at 4 °C overnight. After being washed with PBS, the cells were incubated with corresponding Alexa Fluor 488- or 568-conjugated goat anti-mouse IgG (1:500, Molecular Probes) in the blocking solution at room temperature (23 °C) for 1 hr. In some cases, following the 2^nd^ antibody incubation, the cells were stained by 4′, 6-diamidino-2-phenylindole (DAPI, 0.1 mg/ml, D1306; Molecular Probes) for ~15–20 min to visualize cell nuclei. After washing with PBS, the cells were mounted with a fluorescence mounting medium (H-1000, Vector Lab, CA) and observed under a fluorescence microscope (Nickon, T2000) or a confocal microscope (Leica TCS SP2). The fluorescent image was saved in the TIFF format and assembled in Photoshop (Adobe Systems, CA) for presentation.

### Data analysis

Data were expressed as mean ± s.e.m. unless otherwise indicated in text and plotted by SigmaPlot (SPSS Inc. Chicago, IL). The statistical analyses were performed by SPSS v18.0 (SPSS Inc. Chicago, IL) using one-way ANOVA with a Bonferroni correction or t-test.

## Additional Information

**How to cite this article**: Zong, L. *et al.* Gap junction mediated miRNA intercellular transfer and gene regulation: A novel mechanism for intercellular genetic communication. *Sci. Rep.* **6**, 19884; doi: 10.1038/srep19884 (2016).

## Supplementary Material

Supplementary Figures
