# Supplementary material for: Gap junction mediated miRNA intercellular transfer and gene regulation: A novel mechanism for intercellular genetic communication
Source: Sci Rep. 2016 Jan 27;6:19884. doi: 10.1038/srep19884 (PMC4728487; doi:10.1038/srep19884)
Supplement: Supplementary Figures [file srep19884-s1.pdf]

Supplementary figures:

Gap junction mediated miRNA intercellular transfer and gene regulation: A novel mechanism for intercellular genetic communication

Liang Zong*, Yan Zhu*, Ruqiang Liang, Hong-Bo Zhao

Department of Otolaryngology, University of Kentucky Medical Center, 800 Rose Street, Lexington, Kentucky, USA, 40536

* Equal contributors

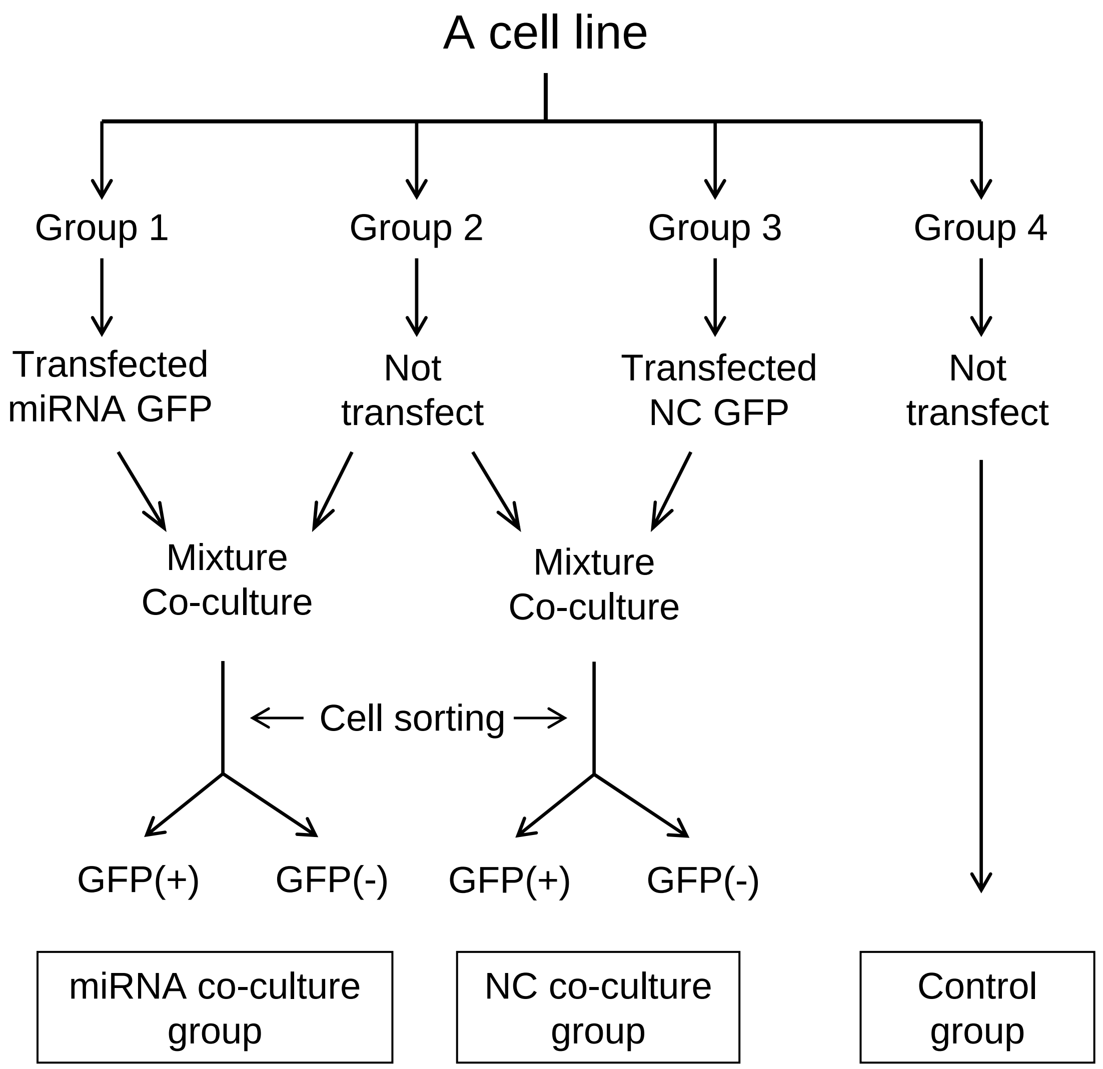

Figure supplement 1

Experimental design and cell co-culture procedure. Each cell line, including the Cx-null cell line, is divided into 4 groups. Two groups are transfected with miRNA GFP vectors and NC GFP vectors, and two other groups have no transfection. Then, transfected cells are mixed with non-transfected cells at 1:1 ratio for co-culture. Non-transfected cells without co-culture serves as a control group.

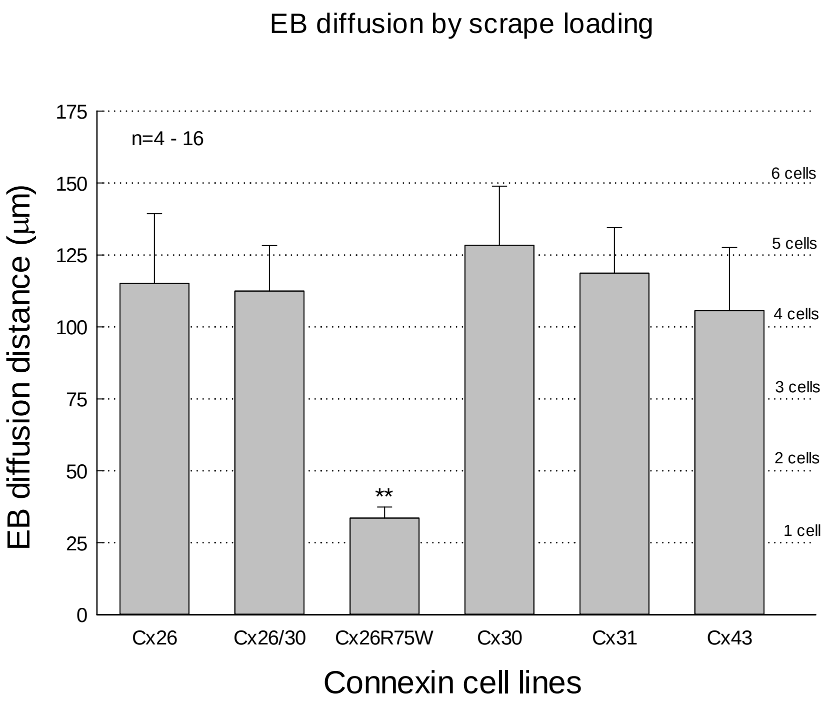

Figure supplement 2

Dye EB diffusion in connexin cell lines assessed by scrape-loading assay. Except the Cx26 R75W mutant cell line, EB diffusion in all tested Cx cell lines reached the 4th cell order and had no significant difference, indicating that these connexin over-expressed cell lines have similar functional expression. In the Cx26 R75W cell line, EB is restricted to the scraped cells (the 1st cell order) and has no labeling in neighboring cells. Data are represented as mean$\pm$SD. **: $P < 0.001$, one-way ANOVA with a Bonferroni correction.
